# Sequential FDG-PET and induction chemotherapy in locally advanced adenocarcinoma of the Oesophago-gastric junction (AEG): The Heidelberg Imaging program in Cancer of the oesophago-gastric junction during Neoadjuvant treatment: HICON trial

**DOI:** 10.1186/1471-2407-11-266

**Published:** 2011-06-24

**Authors:** Sylvie Lorenzen, Carl von Gall, Annika Stange, Georg M Haag, Jürgen Weitz, Uwe Haberkorn, Florian Lordick, Wilko Weichert, Ulrich Abel, Jürgen Debus, Dirk Jäger, Marc W Münter

**Affiliations:** 1 National Center for Tumor Diseases (NCT), University of Heidelberg, Germany; 2 Department of Nuclear Medicine, University of Heidelberg, Germany; 3 Department of Surgery, University Hospital of Heidelberg, Germany; 4 Medizinische Klinik III, Klinikum Braunschweig, Germany; 5 Institute of Pathology, University of Heidelberg, Germany; 6 Department of Medical Biometry, University of Heidelberg, Germany; 7 Department of Radiation Oncology, University of Heidelberg, Germany

## Abstract

**Background:**

18-Fluorodeoxyglucose-PET (^18^F-FDG-PET) can be used for early response assessment in patients with locally advanced adenocarcinomas of the oesophagogastric junction (AEG) undergoing neoadjuvant chemotherapy. It has been recently shown in the MUNICON trials that response-guided treatment algorithms based on early changes of the FDG tumor uptake detected by PET are feasible and that they can be implemented into clinical practice.

Only 40%-50% of the patients respond metabolically to therapy. As metabolic non-response is known to be associated with a dismal prognosis, metabolic non-responders are increasingly treated with alternative neoadjuvant chemotherapies or chemoradiation in order to improve their clinical outcome. We plan to investigate whether PET can be used as response assessment during radiochemotherapy given as salvage treatment in early metabolic non-responders to standard chemotherapy.

**Methods/Design:**

The HICON trial is a prospective, non-randomized, explorative imaging study evaluating the value of PET as a predictor of histopathological response in metabolic non-responders. Patients with resectable AEG type I and II according to Siewerts classification, staged cT3/4 and/or cN+ and cM0 by endoscopic ultrasound, spiral CT or MRI and FDG-PET are eligible. Tumors must be potentially R0 resectable and must have a sufficient FDG-baseline uptake. Only metabolic non-responders, showing a < 35% decrease of SUV two weeks after the start of neoadjuvant chemotherapy are eligible for the study and are taken to intensified taxane-based RCT (chemoradiotherapy (45 Gy) before surgery. ^18^FDG-PET scans will be performed before ( = Baseline) and after 14 days of standard neoadjuvant therapy as well as after the first cycle of salvage docetaxel/cisplatin chemotherapy (PET 1) and at the end of radiochemotherapy (PET2). Tracer uptake will be assessed semiquantitatively using standardized uptake values (SUV). The percentage difference ΔSUV = 100 (SUV_Baseline _- SUV _PET1_)/SUV_Baseline _will be calculated and assessed as an early predictor of histopathological response. In a secondary analysis, the association between the difference SUV_PET1 _- SUV_PET2 _and histopathological response will be evaluated.

**Discussion:**

The aim of this study is to investigate the potential of sequential ^18^FDG-PET in predicting histopathological response in AEG tumors to salvage neoadjuvant radiochemotherapy in patients who do not show metabolic response to standard neoadjuvant chemotherapy.

**Trial Registration:**

Clinical trial identifier NCT01271322

## Background

Oesophageal cancer is among the 10 most common malignancies worldwide and is associated with a high mortality [1,2]. Often, the tumors are locally advanced at the time of initial diagnosis because symptoms do not appear until late (T3-T4, N+, or M1). In cases of locally advanced tumors (T3/T4, N+), surgery remains the mainstay of therapy, but evidence is growing that preoperative chemotherapy or chemoradiotherapy improves survival in responding patients with locally advanced adenocarcinoma of the oesophagus and the oesophagogastric junction [3,4]. However, for patients who do not respond, the prognosis after neoadjuvant therapy might be worse than that of a primarily surgical approach [5]. These metabolic non-responders have a low histopathological response rate of only 5% and a poor prognosis compared with responders [6]. Since about half of the patients treated with neoadjuvant chemotherapy will not respond [7], an early predictor of response would avoid futile therapy and allow patients to pursue other, potentially more efficacious treatments. Therefore, an individual early assessment of response to neoadjuvant therapy using imaging techniques could be of great value for tailoring neoadjuvant treatment as well as the surgical approach to the individual patient [8-10]. Over the past few years, many attempts have been made to improve prognostication of the individual tumor biology in oesophageal carcinoma and to identify prognostic and predictive biomarkers.

Metabolic changes measured by PET have been shown to be more sensitive in detecting response early in the course of chemotherapy as compared with both conventional imaging techniques (EUS and CT) and endoscopy [11]. Various studies have demonstrated that 18-fluorodeoxyglucose-positron emission tomography (FDG-PET), measuring early changes in tumor glucose uptake after only two weeks of induction therapy, is a promising tool in the prediction of clinical and histopathologic response as well as prognosis to neoadjuvant treatment in adenocarcinomas of the oesophagogastric junction (AEG) type I and II [6,12]. Available evidence suggests that metabolic response might be a useful predictive marker for the early identification of non-responding patients. The MUNICON-I trial prospectively showed that early metabolic assessment with therapy stratification after only 2 weeks helps to select patients who are not benefiting from neoadjuvant chemotherapy and can therefore avoid ineffective and toxic therapy in non-responding patients with AEG I and II [8,9]. These patients with poor prognosis and potentially biologically even more aggressive tumors might be suitable candidates for intensification of the neoadjuvant treatment, by changing the chemotherapeutic regimen in attempting to overcome chemoresistance, and by adding radiochemotherapy.

Evidence in the literature gives the rationale to use a taxane-based regimen in patients who are not responding to a first line EOX/EOF (Epirubicin, Oxaliplatin, Capecitabin/5-Fluorouracil) induction therapy [13]. A phase II trial showed that Docetaxel given with Cisplatin is a highly active chemotherapy schedule and permitted surgery in initially inoperable patients [14]. Furthermore, neoadjuvant chemoradiotherapy for adenocarcinoma of the oesophagus seems to improve overall survival [4] and may induce significantly higher response rates compared to chemotherapy alone [15].

The primary objective of the Heidelberg Imaging program in Cancer of the Oesophago-gastric junction during Neoadjuvant treatment (HICON) trial is to investigate whether semiquantitative measurements of ^18^F-FDG accumulation at the primary tumor site using (sequential) PET could be applied to assess the effects of salvage neoadjuvant radiochemotherapy in initially non-responding tumors, with metabolic response defined by a ≥35% decrease in ^18^F-FDG uptake two weeks after induction chemotherapy [6,12], using histopathology as a gold standard.

## Methods/Design

### Study Design

HICON is a prospective, non-randomized, exploratory imaging/biomarker study. The study is designed and coordinated by the National Center for Tumor Disease (NCT), Heidelberg. The protocol of the study was approved by the local Ethics Committee, and was also subject to authorization by the German radiation protection authority (Bundesamt für Strahlenschutz = BfS) as mandatory by federal law.

All participants provide written informed consent.

The study was assigned the number NCT01271322 in the European Clinical Trials Database (EudraCT).

### Study objectives and endpoints

The primary objective of the study is to evaluate the change in metabolic response as measured by the relative difference ΔSUV = 100 (SUV_Baseline _- SUV_PET1_)/SUV_Baseline _in ^18^F-FDG uptake after 1 cycle of salvage taxan-based chemotherapy (PET1), relative to the ^18^F-FDG uptake at the baseline examination, as a predictor of histopathological response in initial metabolic non-responders (assessed by PET 14 days after the start of neoadjuvant therapy). Secondary objectives are the investigation of the distribution of ΔSUV in histological responders and non-responders, the accuracy of the binary prediction rule ΔSUV ≥65% vs. < 65% (in particular the question whether this rule is superior to a random prediction), the association of SUV measured by PET1 with the value of PET response after intensified neoadjuvant radiochemotherapy (PET2), the association between SUV_PET1 _- SUV_PET2 _and histopathological response, and the association between ΔSUV and overall survival as well as disease-free survival (counted from the date of recruitment).

### Patient selection

Eligibility criteria include the presence of biopsy-proven adenocarcinoma of the distal oesophagus (AEG type I) or cardia (AEG type II) [16], staged as cT3 or cT4, with or without metastases in local lymph nodes and no evidence of hematogenous metastases. Staging procedures include endoscopy, endoscopic ultrasound and computed tomography (CT) of the chest and abdomen. Eligible patients have to be fit for platin-containing chemotherapy and tumors must be potentially R0 resectable during consecutive operation.

Tumors must have demonstrated a minimal amount of FDG-uptake in the baseline PET-CT, defined as ^18^FDG-uptake in tumor at first examination > 1,35 × hepatic-SUV + 2 × standard-deviation of hepatic-SUV, and must be a metabolic non-responder under EOX, defined as a decrease of the SUVmax of < 35% in a second PET on day 14 of chemotherapy.

Patients with an Eastern Cooperative Oncology Group score worse than 1, previous or secondary malignancy, life expectancy of less than 3 months, uncontrolled bleeding from the tumor, tumor infiltration of the airways, pregnancy, uncontrolled diabetes, or age less than 18 years are excluded. Patients are also ineligible if they have undergone previous chemotherapy, radiotherapy, or endoscopic laser therapy.

### Treatment schedule/follow-up

After obtaining written informed consent, a baseline FDG-PET will be performed during initial staging within one week before initiation of preoperative chemotherapy. Patients will be eligible for inclusion only if PET scans show a sufficient contrast between tumor and surrounding tissues: this is based on standard uptake value (SUV) measurements. All eligible patients will be given the following induction chemotherapy:

EOX:   Epirubicin 50 mg/m^2 ^i.v. Bolus           d1

           Oxaliplatin 130 mg/m^2 ^i.v.                  d1

           Capecitabin 2 × 625 mg/m^2 ^p.o.         d1-21

Or (only if dysphagia):

EOF:   Epirubicin 50 mg/m^2 ^i.v. Bolus           d1

           Oxaliplatin 130 mg/m^2 ^i.v.                  d1

           5-FU 200 mg/m^2^/d continuously i.v.  d1-21

### FDG-PET will be repeated on day 14 of the first chemotherapy cycle

Patients whose tumor SUV will be decreased by ≥35%, will be defined as metabolic responders. This cut-off is predefined and is based on previous research [6,8,12]. Metabolic responders will continue to receive chemotherapy for a maximum of 12 weeks before surgery.

**Figure 1:**
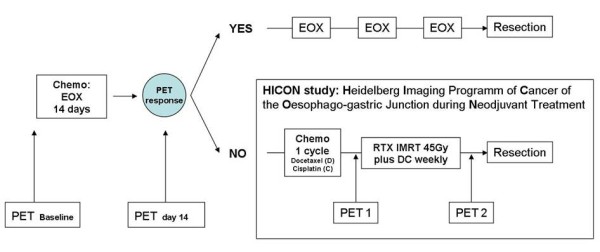
**Trial flow chart**.

Metabolic non-responders, with a SUV decrease of less than 35%, discontinue induction chemotherapy and proceed to an intensified salvage radiochemotherapy treatment, which forms the basis of the HICON study (see Figure 1). Within this imaging/biomarker study, patients will receive two more FDG-PET assessments to evaluate metabolic response: the first right before the onset of radiochemotherapy (PET1) and the second after completion of radiochemotherapy and prior to resection (PET2).

Within the HICON study, patients will be treated with one cycle of chemotherapy with docetaxel 75 mg/m^2^, and cisplatin 75 mg/m^2 ^to allow radiation planning.

This treatment will be followed by radiochemotherapy, starting on day 22 of intensified chemotherapy cycle:

#### Radiotherapy

1.8 Gy/day, total dose 45Gy

#### Docetaxel

25 mg/m^2 ^on days 1, 8, 15, 22, 29 of radiochemotherapy (RCT) and Cisplatin: 25 mg/m^2 ^on days 1, 8, 15, 22, 29 of RCT. Docetaxel should be given before cisplatin.

Chemotherapy is given on a weekly basis. Combined radiochemotherapy, with a total dose of 45Gy, is followed by resection, 28 - 42 days after the end of RCT. The protocol treatment ends 30 days post resection, or if a longer hospital stay is needed, until the last day of hospitalization.

After surgical resection, patients will be followed at 3-month intervals by CT of the chest and abdomen and upper intestinal endoscopy. In patients with curative (R0) resection, time to recurrence will be calculated as the time from initiation of neoadjuvant therapy to detection of local recurrence or distant metastases. In patients with no resection or residual tumor after resection (R1 and R2 resection, respectively), time to tumor progression will be determined.

The duration of the study is anticipated to be 15 months (10 months accrual time plus 5 months of individual follow-up, ending with the assessment of histopathological response). After the end of study participation, patients will be followed according to the standard follow-up scheme implemented at the NCT Heidelberg. Data on survival and relapse gathered during this routine follow-up will be used for the assessment of survival and disease-free survival for study patients.

### Supportive therapy

Proton-pump inhibitors (PPIs) will be given once daily in standard dosage during the entire preoperative period.

Premedication with dexamethasone will be performed according to local standards.

Antiemetic prophylaxis and therapy is recommended according to local standards.

Granulocyte-Colony Stimulating Factor (G-CSF): In the case of neutrophils nadir < 0.5 × 10^9^/l during the previous cycle, G-CSF may be administered during the following cycles according to local standards.

#### Hydration

During CT and RCT, standard i.v. hydration before and after cisplatin treatment is mandatory, according to local rules. Hydration during weekly cisplatin will be done according to local standards.

### PET Imaging

An ^18^F-FDG PET/CT scan will be performed for each patient before the initiation of preoperative chemotherapy (baseline PET) and 14 days after the initiation of chemotherapy (PET day14). In metabolically non-responding patients (< 35% SUV decrease), a third PET scan after initiation of taxan-based chemotherapy and right before radiochemotherapy (PET1) and a fourth PET scan after the completion of radiochemotherapy and immediately before surgery (PET2) will be performed.

PET scans will be done using a Biograph 6 (Siemens AG, Erlangen, Germany), axial field of view of 15.4 cm in 3D mode. Patients will fast at least 6 hours before PET imaging to ensure euglycamic glucose metabolism. Blood glucose levels are measured before each PET scan. Prior to each application, patients are advised to rest and continue to reduce activities for 50 minutes after administration of 250 to 350 MBq 18F-FDG followed by 500 ml saline solution to increase the distribution volume.

60 minutes post injection a whole body scan with 3 min/BP from head to upper femur is performed.

The reconstruction of the raw images is performed after dead time, scatter and random correction using an iterative method based on the ordered subsets expectation maximization algorithm (OSEM) of four iterations/8 subsets, 256 × 256 matrix, Gaussian smoothing and of 3.5 mm transversal slices at full with half maximum. The image data set is normalized for the injected dose and the patients body weight, resulting in parametric imaging using standardized uptake value (SUV) on the basis of the formula "SUV = tissue concentration (Bq/g)/(injected dose (Bq)/ body weight (g))".

The spatial resolution of the reconstructed images is 6 to 8 mm at full width half maximum. For quantitative evaluation, an automated volume of interest (VOI) derived from generated regions of interst (ROI) using the auto3D function within the Syngo Software (Siemens AG, Erlangen, Germany) will be placed over the tumor in the slice with maximum FDG uptake in the baseline scan. In the second PET scan, the region of interest will be placed at the same position as in the baseline PET as a reference. For patients treated in the HICON study, the change of FDG-uptake before (PET1) and after (PET2) radiochemotherapy will be assessed in relation to the baseline PET.

For further analysis the mean value of the two measurements is used.

### Preoperative Radiochemotherapy

The treatment dosage of this radiochemotherapy scheme is based upon a SAKK study (SAKK 75/02), which purpose was to investigate the efficacy, toxicity, and feasibility of preoperative docetaxel-cisplatin together with radiotherapy. This trimodality treatment showed feasibility in combination with favourable antineoplastic activity and survival data compared with other trials [17].

After positioning the patient in an adequate custom-made device a treatment planning CT will be performed. In all cases a contrast-enhanced CT with a thickness of 3 mm is required. The treatment planning CT scan should be acquired with the patient in the same position and using the same immobilization device as for treatment. All tissues receiving irradiation should be included in the CT scan. A slice by slice segmentation of the target volume and all organs at risk will be performed. Gross Tumor Volume (GTV) represents the region judged to contain gross primary tumor or involved node(s) based on CT scan and other imaging techniques. The Clinical Target Volume (CTV) is defined as the GTV plus areas considered at risk for containing microscopic disease delineated by the treating physician. CTV represents the GTV plus a margin of generally 0.2 cm and the nodal regions receiving elective irradiation. The CTV margins can be narrower when GTV is in the proximity of the spinal cord or critical normal tissues. All efforts should be made to adapt and delineate the CTV according to the location of the primary tumor and the relative incidence of lymph node involvement in the different anatomic regions of the lymph nodes. *Planning Target Volume *(PTV) represents an additional margin of at least 0.2 cm around the CTV to compensate for the variability of treatment set up and internal organ motion.

The applied total dose is 45 Gy in 25 fractions (1.8 Gy single dose). Therefore the radio-therapy will be applied in 5 weeks. For the normal tissue the tolerance doses (TD 5/5) defined by Emami et al. [18] will be applied. At least 95% of the CTV should receive 90% of the prescribed total dose and the maximum dose should be smaller than 110%.

For treatment a linear accelerator with at least 6 MeV photons will be used. Intensity modulated radiation therapy (IMRT) is recommended but according to the treatment protocol also 3-D planned treatment techniques are allowed. The linear accelerator should be equipped with image guidance (IGRT) and at least weekly positioning controls will be performed. In case of severe deviations in the positioning of the patients new treatment plans should be calculated.

### Surgical Therapy

The recommended surgical procedure in patients with AEG type I is an abdominothoracic oesophagectomy with intrathoracic anastomosis. In patients with AEG type II tumors, a transhiatal extended gastrectomy and an extended D2-lymphadenectomy, including a left retroperitoneal lymphadenectomy can be performed [19].

### Histopathologic Analysis

For assessment of histopathologic tumor regression, the resected primary tumors will be evaluated according to a recently published scoring system [20]. All patients with less than 10% residual tumor (regression score 1a and 1b) will be classified as responders. All other tumors (regression score 2 and 3) will be classified as nonresponders.

### Statistical considerations and sample size estimation

The sample size/power calculations were based on a univariate logistic regression analysis of the predicitveness of ΔSUV, for histopathological response. The projected sample size of 25 evaluable patients is sufficient to detect, with a power of > 80%, an increase of the AUC of the ROC curve of ΔSUV (with respect to histopathological response) from 0.5 to 0.8, assuming that the values of ΔSUV in the groups R and N are represented by independent normally distributed variables with equal variances, and assuming a histopathological response rate of r = 50% (calculations based on 10000 computer stimulations, α = 10%). While, under the assumptions made above the study was slightly overpowered (estimated power = 84.7%), the sample size of n = 25 to some extent safeguards against the loss of power occurring if r ≠50%, with, e.g., an estimated power of 80.3% resulting for r = 65%. Assuming a drop-out rate of about 10% the total number of patients to be recruited was set at n = 28.

Demographic and other baseline variables, as well as the treatment-related variables (like, e.g., the number of treatment cycles, dosages, dose modifications) will be analyzed using frequency tables in case of categorical variables and summary statistics for quantitative variables. The results of PET scans will be described by means of summary statistics, box plots, and scatter plots, both overall and for histological responders and non-responders separately. The analysis of the primary endpoint will be done using univariate logistic regression with ΔSUV (or a monotone transform of ΔSUV) as the predictor variable and histological response as the outcome variable. In this analysis, the statistical significance level is set at α = 10%. The accuracy of the binary prediciton rule ΔSUV ≥65% vs.< 65% will be estimated together with Clopper-Pearson 95% confidence bounds. Fisher's exact test will be used to test to compare the accuracy of this rule with a random prediction. The association between the difference SUV_PET1 _and SUV_PET2 _will be examined by calculating Spearman's rank correlation coefficient and by analyzing the agreement according to the method proposed by Bland and Altman [21]. The agreement of the results of the aformentioned binary rule applied to both SUV_PET1 _and SUV_PET2 _will be assessed by means of McNemar's test. The association between the difference D12 = SUV_PET1_-SUV_PET2 _and histopathological response will be analyzed by means of the Wilcoxon rank sum test comparing histopathological responders and non-responders. A ROC curve for D12 with respect to response will be produced. The association between ΔSUV and overall survival as well as disease-free survival will be assessed using the Cox Proportional Hazard model. Safety will be analyzed based on adverse evnts and shift tables for laboratory parameters.

Except for the logistic regression analysis of the primary endpoint, all analyses are strictly exploratory. For each particular objective and endpoint, the analysis population consists of all patients for whom the measurements required for the analysis are available. Patients having no PET1-measurement or who drop out before the histopathological response has been determined will be replaced. No interim analyses will be carried out.

## Discussion

The most exciting use of FDG-PET in the management of localized gastroesophageal cancer is the early assessment of metabolic response during neoadjuvant chemotherapy. In this indication, early metabolic response assessment has been shown to contribute to the individualization of treatment algorithms; cut-off values have been prospectively validated and have also been used in interventional clinical studies [6,8,10,22,23].

As metabolically non-responding patients have poor survival chances even after R0 resection, the early identification of these non-responding tumors by FDG-PET may allow an adaption and optimization of preoperative treatment. We hypothesize that non-responding patients after only 2 weeks of chemotherapy might benefit from a change of the chemotherapy regimen and the additional use of radiotherapy.

In previous studies, metabolic non-response was associated with histopathological non-response [8], however it remains to be determined if subsequent radiochemotherapy can transform some metabolic non-responders after induction chemotherapy into histo-pathological responders.

While the utility of FDG-PET for early response assessment has been shown after neoadjuvant chemotherapy alone, the role of FDG-PET in monitoring the antitumoral activity of radiochemotherapy has not been established.

This study was designed to assess the value of metabolic response evaluation before and after initiation of salvage radiochemotherapy (RCT) in patients with metabolic non-response after 2 weeks of induction chemotherapy. In order to overcome chemoresistance, the neo-adjuvant treatment regimen will be intensified by changing the chemotherapeutic agent and adding radiation treatment.

However, this study is only designed as an imaging/biomarker study, and further clinical trials are necessary to eventually answer the question of whether further neoadjuvant chemotherapy or chemoradiation for ^18^F-FDG PET non-responders improves their clinical outcome.

In this context it still needs to be evaluated whether a metabolic response fully captures the net effect of treatment on the clinical outcome.

Only randomized trials will ultimately be able to demonstrate whether treatment regimens with a higher metabolic response rate also lead to improved patient survival.

The European Organization of Research and Treatment of Cancer (EORTC) is currently planning a randomized international multicenter trial, comparing the activity of intensified taxane based RCT to immediate resection in PET non-responders in terms of histopathological response and survival.

The HICON study was designed to prospectively evaluate if quantitative assessment of tumor metabolism by FDG-PET is an efficient and clinically useful technique to monitor tumor response to salvage (radio)-chemotherapy early in the course of treatment.

In conclusion, this study aims to quantify metabolic tumor response by determining whether changes in tumor FDG-uptake, before and after intensified radiochemotherapy can predict histopathological response after completion of neoadjuvant therapy in initially metabolically non-responding patients.

If it would be possible to early select patients who will not benefit from intensified salvage radiochemotherapy, these patients could be spared intensified and potentially toxic salvage treatments which may impair quality of life; alternatively, patients could proceed directly to surgery.

## Competing interests

The authors declare that they have no competing interests.

## Authors' contributions

SL, FL, CvG, AS, MWM, WW, JD, DJ, UH, JW participated in the design of the study, UA was responsible for the statistical planning of the trial, AS and FL wrote the study protocol. SL and GMH are reponsible for conducting and co-ordination of the trial as well as patient recruitment. All authors read and approved the final manuscript.

## Pre-publication history

The pre-publication history for this paper can be accessed here:

http://www.biomedcentral.com/1471-2407/11/266/prepub
